# Transparent Ion-Exchange Membrane Exhibiting Intense Emission under a Specific pH Condition Based on Polypyridyl Ruthenium(II) Complex with Two Imidazophenanthroline Groups

**DOI:** 10.3390/membranes11060400

**Published:** 2021-05-27

**Authors:** Hajime Kamebuchi, Satoshi Tamaki, Atsushi Okazawa, Norimichi Kojima

**Affiliations:** 1 Department of Chemistry, College of Humanities and Sciences, Nihon University, Sakurajosui 3-25-40, Setagaya-ku, Tokyo 156-8550, Japan; 2 Graduate School of Arts and Sciences, The University of Tokyo, Komaba 3-8-1, Meguro-ku, Tokyo 153-8902, Japan; tamaki@mie-takada-hj.ed.jp; 3 Division of Chemistry, Institute of Liberal Education, Nihon University School of Medicine, Oyaguchi Kamimachi 30-1, Itabashi-ku, Tokyo 173-8610, Japan; okazawa.atsushi@nihon-u.ac.jp

**Keywords:** transparent emitter, polypyridyl ruthenium(II) complex, Nafion membrane

## Abstract

The development and the photophysical behavior of a transparent ion-exchange membrane based on a pH-sensitive polypyridyl ruthenium(II) complex, [(bpy)_2_Ru^II^(H_2_bpib)Ru^II^(bpy)_2_](ClO_4_)_4_ (bpy = 2,2′-bipyridine, H_2_bpib = 1,4-bis([1,10]phenanthroline[5,6-*d*]-imidazol-2-yl)benzene), are experimentally and theoretically reported. The emission spectra of [(bpy)_2_Ru^II^(H_2_bpib)Ru^II^(bpy)_2_]@Nafion film were observed between pH 2 and pH 11 and showed the highest relative emission intensity at pH 5 (*λ*_max_^em^ = 594.4 nm). The relative emission intensity of the film significantly decreased down to 75% at pH 2 and 11 compared to that of pH 5. The quantum yields (*Φ*) and lifetimes (*τ*) showed similar correlations with respect to pH, *Φ* = 0.13 and *τ* = 1237 ns at pH 5, and *Φ* = 0.087 and *τ* = 1014 ns and *Φ* = 0.069 and *τ* = 954 ns at pH 2 and pH 11, respectively. These photophysical data are overall considerably superior to those of the solution, with the radiative- (*k*_r_) and non-radiative rate constants (*k*_nr_) at pH 5 estimated to be *k*_r_ = 1.06 × 10^5^ s^−1^ and *k*_nr_ = 7.03 × 10^5^ s^−1^. Density functional theory calculations suggested the contribution of ligand-to-ligand- and intraligand charge transfer to the imidazolium moiety in Ru-H_3_bpib species, implying that the positive charge on the H_3_bpib ligand works as a quencher. The Ru-Hbpib species seems to enhance non-radiative deactivation by reducing the energy of the upper-lying metal-centered excited state. These would be responsible for the pH-dependent “*off-on-off*” emission behavior.

## 1. Introduction

**Figure 1:**
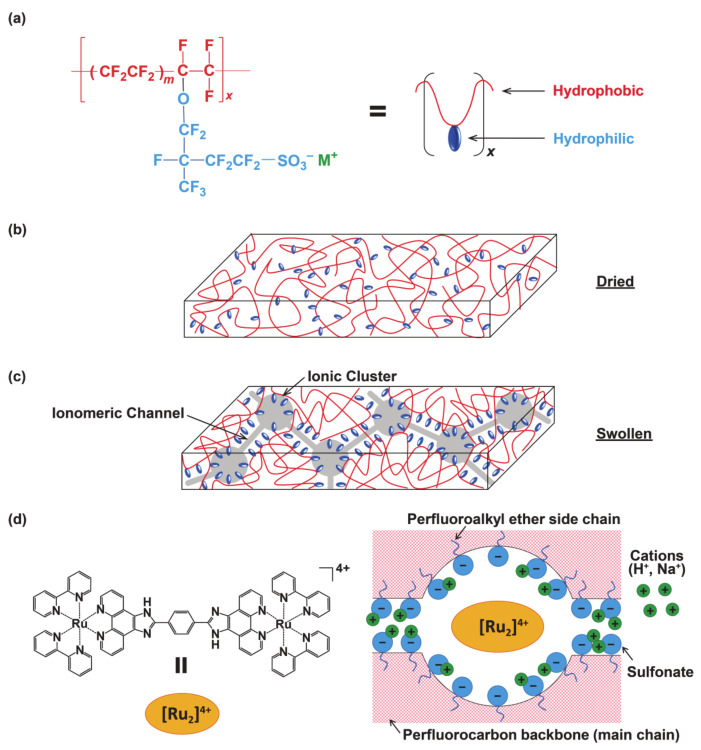
(**a**) Chemical structure of Nafion (*m* = ~6.5). M^+^ is the counter ion such as H^+^, Na^+^. (**b**) Schematic representation of dried and (**c**) swollen membrane morphology. (**d**) Environment of [(bpy)_2_Ru^II^(H_2_bpib)Ru^II^(bpy)_2_]^4+^ in a 5 nm hydrated domain of Nafion. Water or solvent molecules are not shown here for clarity.

In general, transparent light-emitting materials are not visible under room light or sunlight, but are able to emit light upon exposure to a specific wavelength of light, e.g., near-ultraviolet (UV) light, leading to potential applications in various fields such as lighting, displays, security printing, etc. [1]. To obtain the transparent emissive materials, one must prepare nanometer-sized particles that do not scatter visible wavelengths like quantum dots (2–10 nm) [2,3], or otherwise one must disperse molecular emitters into a transparent matrix [4,5,6,7]. In particular, flexible polymer matrices have recently attracted much attention since they provide solid materials in which the shapes such as thickness or dimensions can be easily controlled. Among a number of polymeric materials, the cation exchange membrane Nafion possesses excellent durability on chemical and mechanical aspects, and its large ion-exchange capacity allows us to conventionally obtain highly emissive transparent films [8,9,10,11,12,13,14,15,16,17]. Nafion possesses a perfluorocarbon backbone with a perfluoroalkyl ether side chain that terminates in a sulfonic acid group (Figure 1) [18,19,20,21,22]. Due to the high electron-withdrawing property of the perfluoroalkyl group, proton dissociation occurs readily upon hydration of the Nafion membrane and leads to sulfonate anion (-SO_3_^−^) formation. This means that Nafion is hydrophilic and has a high affinity for protons. If a cationic coordination compound is incorporated, the -SO_3_^−^ group works as a counter anion and the original anion (ClO_4_^−^ in this study) is not allowed to get into the Nafion. The internal structure of Nafion has been inferred mainly by small-angle X-ray scattering techniques [22]. The sulfonate anions create hydrophilic nanopores with a diameter of ~4 nm, in which water molecules and protons are assembled to form clusters, and each cluster is interconnected by hydrophilic channels. The process of “swelling” was experimentally clarified by surface plasmon resonance and neutron reflectometry [23]. Owing to the nanopores in the swollen state, organic compounds and coordination compounds can be transferred to the interior of Nafion by simply soaking the membrane in a solution. Accordingly, transparent films with light-emitting, magnetic, or photo-functional properties can be easily obtained by using ion-exchange membranes such as Nafion without spin coating or casting, which are inevitable parts of constructing composites using conventional polymers [24,25,26,27,28,29,30,31,32].

Proton-responsive transparent emissive materials are in their element when it comes to visualizing pH. For example, the activities of enzymes, cells, bacteria, etc. are often enhanced at a specific pH and may not work well beyond that pH range. Chan et al. have demonstrated that a composite of polypyridyl-ruthenium(II) complex [Ru^II^(bpy)_2_(dhphen)]^2+^ (bpy = 2,2′-bipyridine, dhphen = 4,7-dihydroxy-1,10-phenanthroline) and Nafion works as a pH sensor [15]. The film [Ru^II^(bpy)_2_(dhphen)]@Nafion shows a continuous decrease in metal-to-ligand charge transfer (MLCT) absorption and emission intensities from pH 1 to 8 due to the proton dissociation of the -OH group, which enables us to recognize the pH. This behavior has also been applied to pH monitoring of fermentation by *Klebsiella pneumoniae* [14]. The luminophore is relatively easy to synthesize, and common polypyridyl-ruthenium(II) complexes including [Ru^II^(bpy)_3_]^2+^ are used in life sciences since they are regarded to be non-toxic [33]. The applicable pH range, unfortunately, is rather limited with a low relative emission intensity in the neutral region. Now, let us consider the importance of being able to sensitively recognize pH changes near neutral. The pH of normal cells is usually around 7.4, while it is known that it shifts to the acidic side of 6.5 in human cancer tissues [34]. In other cases, most common bacteria grow optimally around pH 7 and are less able to survive if the pH value is varied by one or two [35,36,37,38]. Health hazards may occur with exposure to conditions other than neutral in a work environment where chemicals are handled. With such backgrounds, the development of a transparent emissive film that is sensitive to pH around neutral has been decided. A polypyridyl ruthenium(II) complex with two imidazole moieties, [(bpy)_2_Ru^II^(H_2_bpib)Ru^II^(bpy)_2_](ClO_4_)_4_·3H_2_O (H_2_bpib = 1,4-bis([1,10]phenanthroline[5,6-*d*]-imidazol-2-yl)benzene) (Figure 1d), was employed as a promising candidate [39,40]. The H_2_bpib ligand and related imidazophenanthrolines can form homo- and hetero-binuclear complexes with Ru, Rh, and Ir and work as a stimuli-responsive luminescent material [41,42,43,44,45,46,47,48,49,50,51,52,53,54]. The complex [(bpy)_2_Ru^II^(H_2_bpib)Ru^II^(bpy)_2_]^4+^ shows a pH-dependent emission intensity from the ^3^MLCT excited state, which is particularly strong in the pH range of 5.9–8.5 [39]. Immobilization of [(bpy)_2_Ru^II^(H_2_bpib)Ru^II^(bpy)_2_]^4+^ as a luminophore in a Nafion membrane should yield a desirable transparent emissive film.

This paper describes the preparation, emission spectra, and photophysical properties of the pH-responsive transparent film [(bpy)_2_Ru^II^(H_2_bpib)Ru^II^(bpy)_2_]@Nafion. The high positive charge of [(bpy)_2_Ru^II^(H_2_bpib)Ru^II^(bpy)_2_]^4+^ in the solution allows it to be spontaneously transferred to the interior of Nafion and immobilized owing to its cation exchange properties. The MLCT emission intensity, quantum yield (*Φ*), and lifetime (*τ*) of the film are well enhanced in the neutral region. In addition, density functional theory (DFT) calculations provided an insight into the pH dependence of the emission intensity of [(bpy)_2_Ru^II^(H_2_bpib)Ru^II^(bpy)_2_]^4+^.

## 2. Materials and Methods

### 2.1. General Procedures

All reagents and solvents were commercially available and used without further purification. The ruthenium(II) dinuclear complex, [(bpy)_2_Ru^II^(H_2_bpib)Ru^II^(bpy)_2_](ClO_4_)_4_·3H_2_O, was synthesized according to [40]. Nafion 117, produced by DuPont (Wilmington, DE, USA), was purchased from Furukawa Agency Co., Ltd. (Tokyo, Japan). UV-vis absorption spectra were recorded using a V-530 spectrophotometer (JASCO Co., Ltd., Tokyo, Japan). Emission spectra were recorded on an F-4500 Fluorescence Spectrometer (Hitachi High-Technologies Co., Ltd., Tokyo, Japan). Emission quantum yields and lifetimes were measured using a Quantaurus-QY absolute PL quantum yield spectrometer and a Quantaurus-Tau fluorescence lifetime spectrometer (Hamamatsu Photonics K.K., Shizuoka, Japan), respectively.

### 2.2. Preparation of the Films

The commercially available Nafion 117 membrane (15W × 40D × 0.187t mm^3^) was soaked in an aqueous solution of NaOH (2 M) for 24 h to replace the cations retained within Nafion with Na^+^ ions (Na-form). Then, [(bpy)_2_Ru^II^(H_2_bpib)Ru^II^(bpy)_2_](ClO_4_)_4_·3H_2_O in MeOH (2.70 × 10^−4^ M, 2 mL) was combined with Britton–Robinson buffer (10 mL; pH 2, 5, 8, 11) [55]. Soaking the Na-form Nafion in the pH-adjusted mixture for 24 h at room temperature yielded the transparent films [(bpy)_2_Ru^II^(H_2_bpib)Ru^II^(bpy)_2_]@Nafion.

### 2.3. DFT Calculation

DFT calculations were performed in the gas phase using the Gaussian 09 (Revision D.01) program package [56]. The ground-state molecular geometry was optimized by using B3LYP functional [57,58] and 6-31G(d,p) basis set [59] on all atoms except for ruthenium where the Stuttgart/Dresden (SDD) pseudopotential [60] was used to treat the metal electronic core, and the metal valence electrons were treated using the SDD basis set. GaussView 5.0.9 was used to visualize the input and the output files.

## 3. Results and Discussion

### 3.1. Determination of the Loaded Molecules into Nafion by UV-Vis Spectroscopy

**Figure 2:**
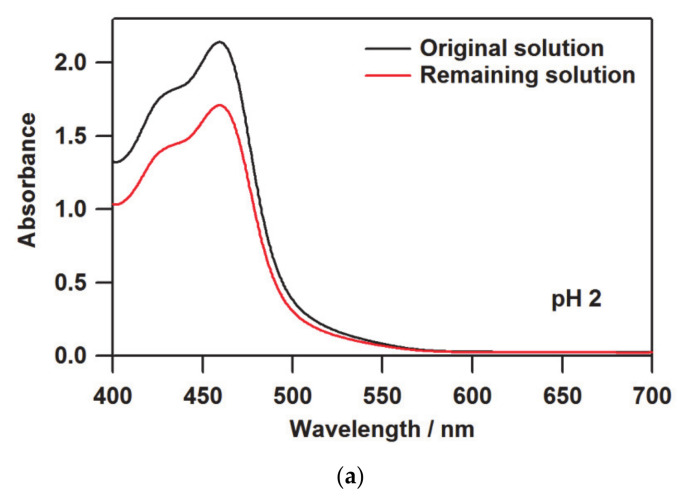

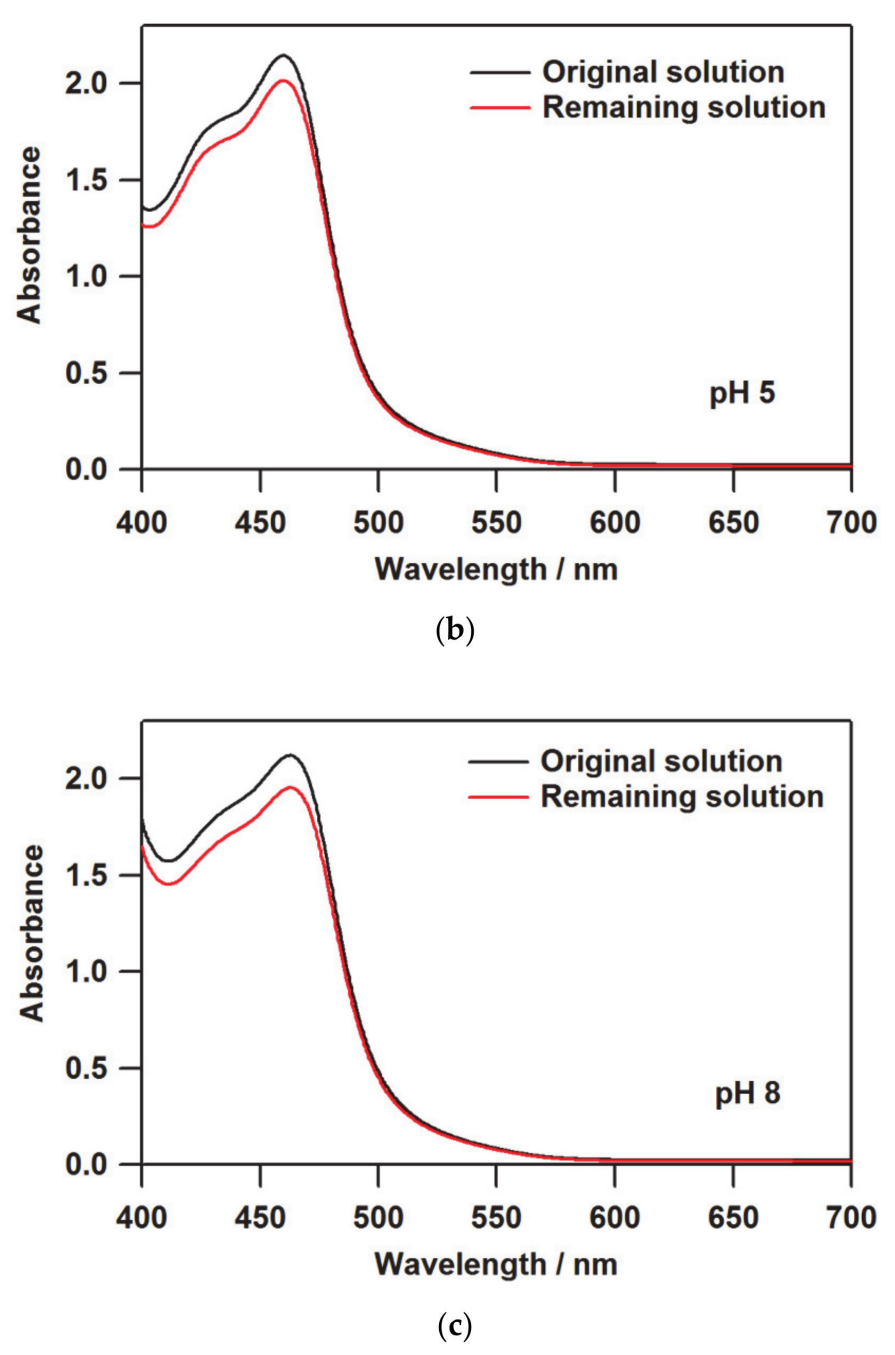
UV-vis spectra before and after soaking the Na-form Nafion membrane into the solution of [(bpy)_2_Ru^II^(H_2_bpib)Ru^II^(bpy)_2_](ClO_4_)_4_·3H_2_O at (**a**) pH 2, (**b**) 5, and (**c**) 8. The absorbance of the remaining solution is observed to be lower than that of the original solution, and the difference corresponds to the number of molecules of the ruthenium(II) complex loaded into Nafion.

In the process of preparing the films using cation exchange by Nafion, the amount or concentration of the molecules loaded into the film is not obvious, unlike the spin coating and casting methods. Hence, UV-vis absorption spectroscopy was employed to determine the amount of molecules introduced into the Nafion membrane. Figure 2 shows the UV-vis absorption spectra before and after soaking the Na-form Nafion membrane into the [(bpy)_2_Ru^II^(H_2_bpib)Ru^II^(bpy)_2_](ClO_4_)_4_·3H_2_O solution. Since the ruthenium(II) complex has p*K*_a_ = 4.11 and 7.84 [39], [(bpy)_2_Ru^II^(H_3_bpib)Ru^II^(bpy)_2_]^5+^ would be the main species upon exposure to the pH 2 environment. Even though the title complex is indeed large in size, it is an intrinsic property of Nafion that highly charged materials facilitate the ion exchange. In addition, a reported case has demonstrated that [{(bpy)_2_Ru(dpp)}_2_RhBr_2_]^5+^ (dpp = 2,3-bis(2-pyridyl)pyrazine) is transported into Nafion when a solvent with high swelling ability for Nafion is used [61]. Considering the charge balance, this means that five M^+^ ions were replaced with a [(bpy)_2_Ru^II^(H_3_bpib)Ru^II^(bpy)_2_]^5+^ molecule to immobilize it in Nafion by ion exchange leading to a decrement of the concentration of the complex solution. This is observable as a difference in absorbance in the UV-vis spectrum, which can be used to estimate the number of molecules loaded into Nafion. According to the Beer–Lambert law, the following relationship is established between absorbance (*A*), molar extinction coefficient (*ε*), molar concentration of the solution (*c*), and optical path length (*l*):

*A* = *εcl* (1)

**Figure 3:**
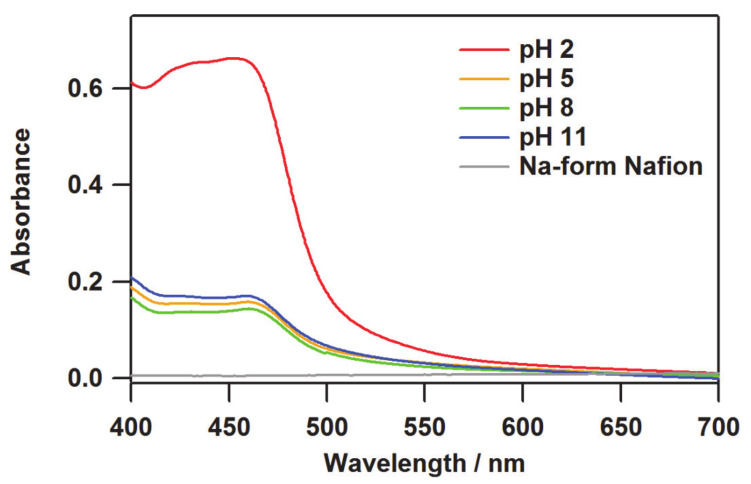
The pH dependence of UV-vis absorption spectra on [(bpy)_2_Ru^II^(H_2_bpib)Ru^II^(bpy)_2_]@Nafion films.

Let us define the absorbance of the original and the remaining solutions as *A*_0_ and *A*, respectively, then the ratio between them is:

(2) $$\frac{A}{A_{0}}=\frac{\varepsilon cl}{\varepsilon c_{0}l}=\frac{c}{c_{0}}$$

where *c*_0_ and *c* are the concentrations of the original and the remaining solutions, respectively. Accordingly, *c* can be estimated from *A*/*A*_0_, where the difference between *c*_0_ and *c* can be regarded as the amount loaded into Nafion. The absorbances at the maximum absorption wavelength of 459.5 nm were *A*_0_ = 2.140 and *A* = 1.708, and the number of molecules loaded into Nafion was estimated to be 1.088 × 10^−7^ mol. This corresponds to 4.429 × 10^−7^ mol per 1 g of Nafion and 0.049% of the counter cation replaced by [(bpy)_2_Ru^II^(H_3_bpib)Ru^II^(bpy)_2_]^5+^ per -SO_3_^−^ group, based on the equivalent weight of Nafion as 1100 g mol^−1^ [20]. In the cases of pH 5 and 8, the amounts loaded were 1.328 × 10^−7^ mol g^−1^ (0.015%) and 1.719 × 10^−7^ mol g^−1^ (0.019%), respectively, which are summarized in Table 1. The difference in absorbance ratio *A*/*A*_0_ at pH 5 and 8 is just 0.018. This rather small difference would include experimental errors related to solution preparation or absorption intensity collection. We consider that the amounts loaded at pH 5 and 8 are almost consistent and the difference is not meaningful. The smaller amount loaded compared to the pH 2 film is likely due to the reduction in positive charge at pH 5 and 8, where [(bpy)_2_Ru^II^(H_2_bpib)Ru^II^(bpy)_2_]^4+^ is the main species. The same method could not be used to estimate the exact amount loaded at pH 11 because, unfortunately, a small amount of precipitation occurred in the solution during the ion exchange for 24 h. Nevertheless, the absorption intensity of the film at pH 11 is comparable to those at pH 5 and 8 as can be seen in the UV-vis spectra of [(bpy)_2_Ru^II^(H_2_bpib)Ru^II^(bpy)_2_]@Nafion shown in Figure 3. The absorption intensity at 380 nm (*I*(380)) in [(bpy)_2_Ru^II^(H_2_bpib)Ru^II^(bpy)_2_]^4+^ is considered to be an isosbestic point independent of pH [39]; in fact, the absorbance at pH 5, 8, and 11 is *I*(380) = 0.223, 0.198, and 0.246, respectively. Consequently, a similar number of [(bpy)_2_Ru^II^(H_2_bpib)Ru^II^(bpy)_2_]^4+^ molecules must have been loaded into the film at pH 11. The overall higher absorption intensity in the film at pH 2 is due to the greater amount of the ruthenium(II) complex included in Nafion. The normalization of the emission spectrum described in the next section can be done based on the value of *I*(380). The absorption bands at 420–460 nm can be attributed to Ru → bpy and Ru → H_2_bpib MLCTs, which will be discussed in the section on DFT results.

### 3.2. Emission Spectroscopy and Photophysical Property of the Film

**Figure 4:**
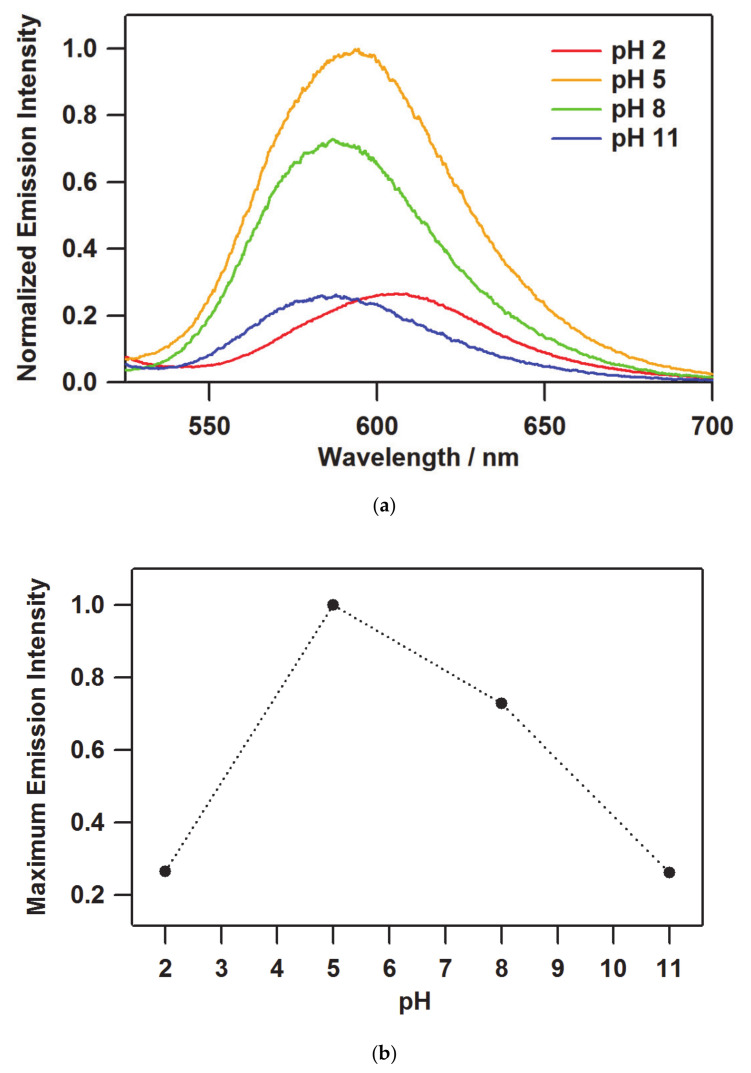
The pH dependence of (**a**) the emission spectra and (**b**) the maximum emission intensity of [(bpy)_2_Ru^II^(H_2_bpib)Ru^II^(bpy)_2_]@Nafion.

In the case of the emission spectra of [(bpy)_2_Ru^II^(H_2_bpib)Ru^II^(bpy)_2_]@Nafion films, the intensity depends in part on the number of the luminescent molecules included in the film as well as the pH. The original emission spectra were normalized by the value of *I*(380) in Table 1 to evaluate only the pH dependence of the emission intensity, and further normalized with respect to the maximum intensity (*λ*_max_^em^) at pH 5 as shown in Figure 4a. A relatively weak emission intensity was observed at pH 2, with an intensity of 0.265 at *λ*_max_^em^ = 608.2 nm. In the pH range where [(bpy)_2_Ru^II^(H_2_bpib)Ru^II^(bpy)_2_]^4+^ is the main species, a strong phosphorescence from the MLCT excited state would be observed. Actually, a significant enhancement of the emission intensity with increasing pH value was observed at pH 5, with the highest relative intensity at *λ*_max_^em^ = 594.4 nm. After a slight decrease in intensity at pH 8, it showed a significant drop at pH 11 resulting in the lowest relative intensity of 0.263 at *λ*_max_^em^ = 587.6 nm. As can be seen in the pH dependence of the maximum emission intensity (Figure 4b), the enhancement of 75% was observed in the film at pH 5 compared to pH 2 and 11. Accordingly, the functional transparent film remarkably indicative of near-neutral pH has been successfully obtained and evaluated.

**Figure 5:**
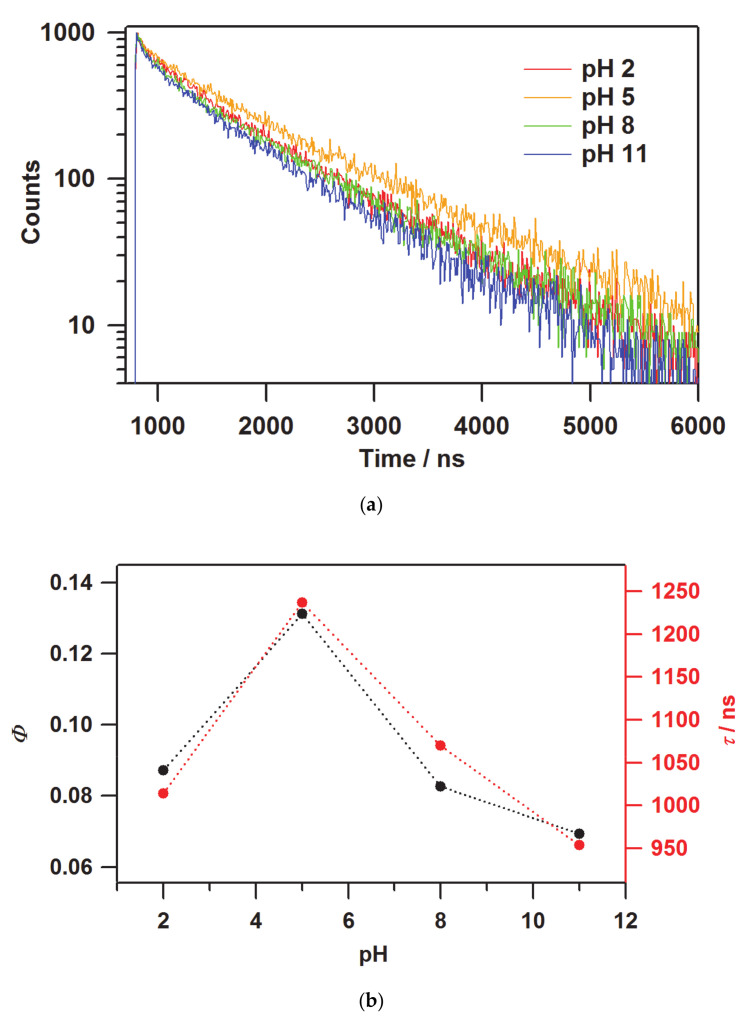
(**a**) The emission lifetime decay curves and (**b**) the values of *Φ* and *τ* (long component) of [(bpy)_2_Ru^II^(H_2_bpib)Ru^II^(bpy)_2_]@Nafion films as a function of pH.

The photophysical properties of quantum yields and lifetimes in [(bpy)_2_Ru^II^(H_2_bpib)Ru^II^(bpy)_2_]@Nafion films were investigated to physicochemically characterize their emission properties. The values obtained are summarized in Table 2. The emission quantum yield of the film at pH 5 was still the maximum value of *Φ* = 0.131. Both extremes at pH 2 and 11 resulted in *Φ* = 0.087 and 0.069, respectively. These results are in agreement with the pH dependence of the emission intensity. [(bpy)_2_Ru^II^(H_2_bpib)Ru^II^(bpy)_2_](PF_6_)_4_ in acetonitrile (MeCN) has been reported to show *Φ* = 0.057 [62], suggesting that the overall quantum yields were enhanced by processing to the film state. This is an important advantage of transparent light-emitting materials. In terms of the emission lifetime profile, the decay curves are shown in Figure 5a. The best-fit results for the decay curves at all pH conditions were obtained by fitting with two components. The short components are *τ* ~200 ns for all the pH conditions, which is much shorter than the lifetime of MLCT emission shown by typical [Ru(bpy)_3_](PF_6_)_2_ of 860 ns and hence cannot be easily assigned [63]. This might come from some sort of metastable species related to the stabilization process of [(bpy)_2_Ru^II^(H_2_bpib)Ru^II^(bpy)_2_]^4+^ in the excited state. Such a shorter lifetime component has also been observed in [Ru(bpy)_3_]@Nafion [64]. The long lifetime (main) component, on the other hand, is *τ* ~ 1000 ns or longer, in which the pH dependence also shows a similar behavior to that of the emission intensity and quantum yield (Figure 5b). A considerable enhancement was observed in the [(bpy)_2_Ru^II^(H_2_bpib)Ru^II^(bpy)_2_]@Nafion film at pH 5 as *τ* = 1060 ns has been reported previously for [Ru(bpy)_3_](PF_6_)_2_ in MeCN [62]. It has long been accepted that the lifetime could be enhanced upon immobilization of an emissive coordination compound such as [Ru(bpy)_3_]^2+^ in a solid matrix like a polymer or glass [65]. This is mainly due to the reduction of oxygen quenching and molecular motion in the excited state. Actually, it has been reported that the lifetime of [Ru(bpy)_3_]@PMMA (PMMA = polymethyl methacrylate) can be enhanced to *τ* = 1500 ns [66]. The reduction in the contribution of non-radiative deactivation as a result of the suppression of molecular motion in the excited state is a key factor in these cases. It would also be related to the possibility of reducing the effect of concentration quenching. This is an important topic that has been demonstrated in other groups as well as in ours as “Rigid Medium Effects” [9,67,68,69]. The rate constants of radiative (*k*_r_) and non-radiative (*k*_nr_) deactivations in [(bpy)_2_Ru^II^(H_2_bpib)Ru^II^(bpy)_2_]@Nafion film at pH 5 were estimated to be *k*_r_ = 1.06 × 10^5^ s^−1^ and *k*_nr_ = 7.03 × 10^5^ s^−1^ based on the experimentally obtained values of *Φ* and *τ*. As can be seen from the previously reported [Ru(bpy)_3_](PF_6_)_2_ values of *k*_r_ = 0.97 × 10^5^ s^−1^ and *k*_nr_ = 9.2 × 10^5^ s^−1^ in MeCN [70], the *k*_r_ of [(bpy)_2_Ru^II^(H_2_bpib)Ru^II^(bpy)_2_]@Nafion is almost comparable, while the decrease in *k*_nr_ is remarkable. As it relates to the rigid medium effects, the following can also be noted. The maximum absorption wavelengths of the film in Figure 3 are 451, 459.5, 460.5, and 459 nm for pH 2, 5, 8, and 11, which are consistent with the previous case [39]. Then, the maximum emission wavelength of 608.2 nm at pH 2 in Figure 4a is in agreement with the literature [39], but the blue shifts at conditions above pH 5 have not been observed in solution. A blue shift not observed in solution is the characteristic behavior of luminophores in matrices, and these were demonstrated in the references [9,67,68,69].

### 3.3. DFT Calculations

**Figure 6:**
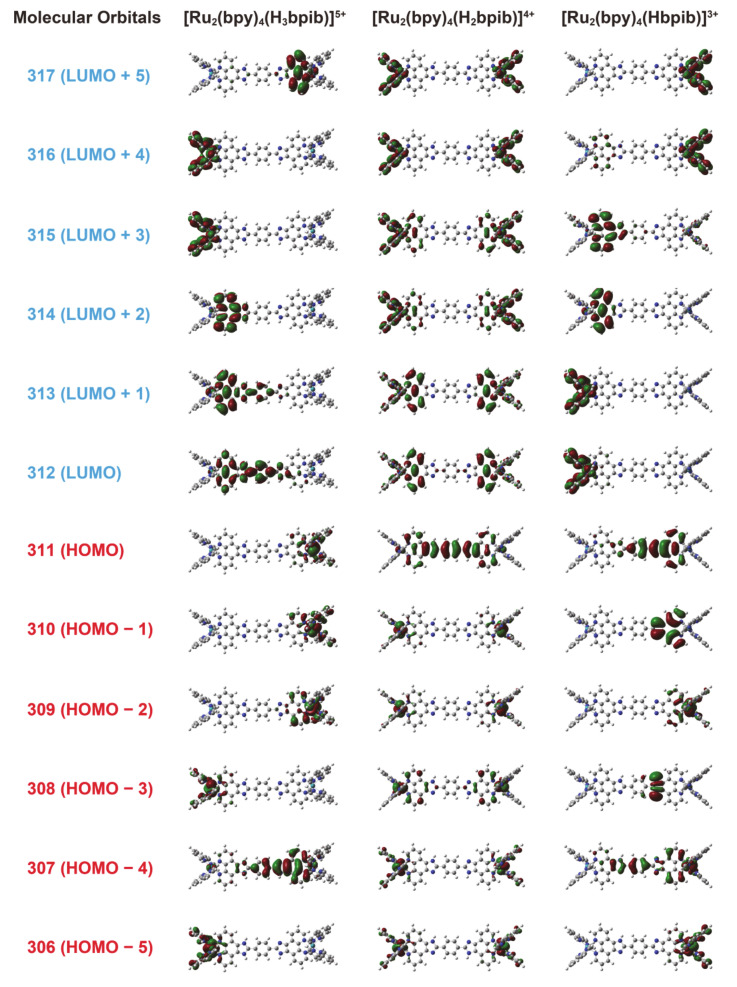
DFT-calculated molecular orbitals of [(bpy)_2_Ru^II^(H_3_bpib)Ru^II^(bpy)_2_]^5+^, [(bpy)_2_Ru^II^(H_2_bpib)Ru^II^(bpy)_2_]^4+^, and [(bpy)_2_Ru^II^(Hbpib)Ru^II^(bpy)_2_]^3+^ at the B3LYP/6-31G**/SDD(Ru) level of theory.

**Figure 7:**
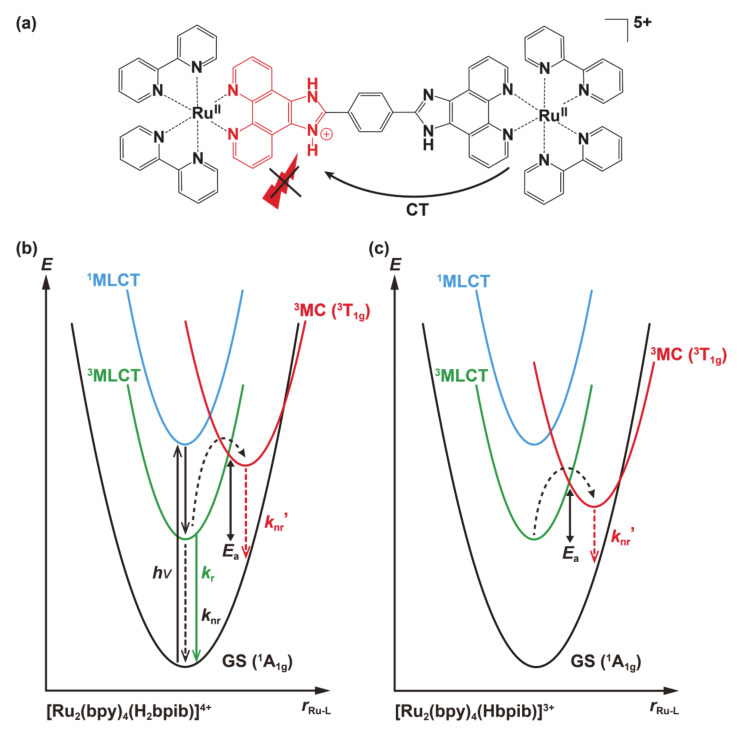
Overview of the possible “*off-on-off*” emission behavior suggested by the DFT calculations. (**a**) [Ru_2_(bpy)_4_(H_3_bpib)]^5+^; Emission caused by the charge transfer to the imidazolium moiety would be quenched due to the positive charge. (**b**) [Ru_2_(bpy)_4_(H_2_bpib)]^4+^; Schematic diagram of a potential energy surface similar to common polypyridyl-ruthenium(II) complexes. (**c**) [Ru_2_(bpy)_4_(Hbpib)]^3+^; The upper-lying ^3^MC excited state becomes more thermally accessible from the ^3^MLCT excited state due to a reduced activation energy (*E*_a_), which causes an increase in *k*_nr_′.

The molecular orbitals obtained from the structural optimization of [(bpy)_2_Ru^II^(H_3_bpib)Ru^II^(bpy)_2_]^5+^, [(bpy)_2_Ru^II^(H_2_bpib)Ru^II^(bpy)_2_]^4+^, and [(bpy)_2_Ru^II^(Hbpib)Ru^II^(bpy)_2_]^3+^ by DFT calculations provided an insight into the origin of the “*off-on-off*” behavior of the emission properties (Figure 6). The summarized possible mechanism is shown in Figure 7. The highest occupied molecular orbital (HOMO) of [(bpy)_2_Ru^II^(H_2_bpib)Ru^II^(bpy)_2_]^4+^ is well distributed on Ru atoms including down to HOMO-5. Looking at the lowest unoccupied molecular orbital (LUMO) or the higher energy orbitals, the distribution of electrons in the bpy ligand and in the phenanthroline moiety of H_2_bpib confirms the existence of the MLCT excited state. Then, the HOMO to HOMO-2 of [(bpy)_2_Ru^II^(H_3_bpib)Ru^II^(bpy)_2_]^5+^, where one imidazole ring is protonated, is distributed on the neutral imidazophenanthroline-Ru(bpy)_2_ side. On the contrary, LUMO to LUMO+4 implies electron transfer to the imidazolium side or bpy ligand. This indicates a slight reduction in the contribution of the emissive MLCT excited states and a mixing of the ligand-to-ligand charge transfer (LLCT) and intraligand charge transfer (ILCT) excited state contributions. It is also known from a previous study of [Ru^II^(bpy)_2_(R_2_bpy)](PF_6_)_2_ (R = methylene amines) that positively charged species by protonation tend to quench the emission because they strongly accept electrons [71]. On the other hand, the HOMO of the singly deprotonated [(bpy)_2_Ru^II^(Hbpib)Ru^II^(bpy)_2_]^3+^ is found to be distributed around the imidazolate, and the distributions above the LUMO can be seen in the Ru(bpy)_2_ fragment or the neutral imidazophenanthroline moiety. This also suggests the contribution of the LLCT and ILCT excited states as well as the emissive MLCT ones. It is also important to note that the deprotonated imidazolate moiety is negatively charged. It decreases the π-acceptance of the ligand, resulting in a higher energy of the *t*_2g_ orbitals and a concomitant decrease in the ligand-field strength. Since the energy of the metal-centered (MC) excited state (*t*_2g_^5^*e*_g_) depends on the ligand field strength, this implies a lower energy of the upper-lying MC state. The lowest excited state of a common emissive ruthenium(II)-polypyridyl compound is ^3^MLCT (or ^3^LC), and it acquires strong emission by making the energy of the MC excited state much higher than that of MLCT by greater ligand-field strength [72]. If the energy of the MC excited state decreases, it becomes more thermally accessible to the upper-lying MC state from the MLCT state and immediately goes into non-radiative deactivation. As described above, the emission of Ru-H_3_bpib and Ru-Hbpib species can be quenched in the process of protonation/deprotonation with respect to Ru-H_2_bpib species in the neutral pH range. In any case, the H_2_bpib ligand not only provides bimetallic coordination compounds, but the emission properties are responsive to both protonation and deprotonation with quite a wide pH range.

## 4. Conclusions

Development of the transparent light-emitting film by fusion of the ion-exchange membrane Nafion with the polypyridyl-ruthenium(II) complex [(bpy)_2_Ru^II^(H_2_bpib)Ru^II^(bpy)_2_](ClO_4_)_4_·3H_2_O (bpy = 2,2′-bipyridine, H_2_bpib = 1,4-bis([1,10]phenanthroline[5,6-*d*]-imidazol-2-yl)benzene) and the investigation of its emission properties have been described. The transparent emissive film [(bpy)_2_Ru^II^(H_2_bpib)Ru^II^(bpy)_2_]@Nafion was obtained by soaking the membrane in the solution of the cationic Ru^II^ complex, owing to the cation exchange property of Nafion. The number of the coordination compounds immobilized on the interior of Nafion was estimated by UV-vis spectroscopy as ~10^−7^ mol g^−1^. Regarding the pH dependence of the emission spectra of [(bpy)_2_Ru^II^(H_2_bpib)Ru^II^(bpy)_2_]@Nafion, the maximum emission intensity was observed at pH 5, and the relative intensities at pH 2 and pH 11 were 0.265 and 0.263, respectively. The pH dependence of the emission quantum yields (*Φ*) and lifetimes (*τ*) was also in accordance with that of the intensity (*Φ* = 0.069–0.131 and *τ* = 954–1237 ns (long component)). Molecular orbitals obtained from the density functional theory calculations provided some insight into the origin of such pH dependence. The positively charged sites of the protonated Ru-H_3_bpib species would behave as a quencher, while the deprotonated Ru-Hbpib species would also be quenched by the approaching energy difference between the emissive metal-to-ligand charge transfer and the metal-centered excited states. In any case, these photophysical data values are still superior to those in solution due to the significant suppression of non-radiative deactivation by the establishment of the film. This study has demonstrated the usefulness of ion-exchange membranes for transparent light-emitting materials. This kind of transparent film, which emphasizes a specific pH will expand its potential applications beyond lighting or displays to other fields such as life sciences.

## Figures and Tables

**Table 1:** Summary of the quantitative determination on [(bpy)_2_Ru^II^(H_2_bpib)Ru^II^(bpy)_2_]@Nafion by UV-vis spectroscopy.

pH	*A*/*A*_0_ ^a^	Loaded Quantity/10^−7^ mol g^−1 b^	Ratio/% ^c^	*I*(380) ^d^
2	0.798	4.429	0.049	0.755
5	0.939	1.328	0.015	0.223
8	0.921	1.719	0.019	0.262
11	-	-	-	0.246

^a^ The ratio of absorbance at the maximum absorption wavelength for the original and the remaining solutions; ^b^ The number of molecules per 1 g of Nafion loaded into the film; ^c^ Percentage of [(bpy)_2_Ru^II^(H_2_bpib)Ru^II^(bpy)_2_] relative to the number of sulfonic acid groups in Nafion; ^d^ The UV-vis absorption intensity of [(bpy)_2_Ru^II^(H_2_bpib)Ru^II^(bpy)_2_]@Nafion at 380 nm.

**Table 2:** Photophysical data of [(bpy)_2_Ru^II^(H_2_bpib)Ru^II^(bpy)_2_]@Nafion films.

pH	*λ*_max_^em^/nm	Max. Intensity	*Φ*	*τ*/ns	*k*_r_ ^a^/10^5^ s^−1^	*k*_nr_ ^b^/10^5^ s^−1^
2	608.2	0.265	0.087	2,521,014	0.86	9.00
5	594.4	1	0.131	2,681,237	1.06	7.03
8	586.4	0.729	0.083	1,991,070	0.77	8.57
11	587.6	0.263	0.069	208,954	0.73	9.76

(a) *k*_r_ = *Φ*/*τ*; (b) *k*_nr_ = *k*_r_(1 − *Φ*)/*Φ.*
